# Psychometric properties of Persian version of diabetes health literacy scale (DHLS) in patients with type 2 diabetes

**DOI:** 10.1186/s13098-022-00910-0

**Published:** 2022-09-27

**Authors:** Mahdi Moshki, Ali Alami, Zohreh Zadehahmad, Mousa Ghelichi-Ghojogh, Mitra Dogonchi, Alireza Jafari

**Affiliations:** 1 Department of Health Education and Health Promotion, School of Health, Social Development and Health Promotion Research Center, Gonabad University of Medical Sciences, Gonabad, Iran; 2 Department of Epidemiology and Bio-Statistics, School of Public Health, Social Determinants of Health Research Center, Gonabad University of Medical Sciences, Gonabad, Iran; 3 Department of Public Health, School of Health, Torbat Heydariyeh University of Medical Sciences, Torbat Heydariyeh, Iran; 4 Health Management and Social Development Research Center, Faculty of Health, Golestan University of Medical Sciences, Gorgan, Iran

**Keywords:** Psychometric, Validity, Reliability, Diabetes health literacy scale, DHLS, Type 2 diabetic

## Abstract

**Background:**

The purpose of this study was to investigate the psychometric properties of the Persian version of Diabetes Health Literacy Scale in type 2 diabetic patients.

**Method:**

This cross-sectional study was conducted in 2021 in 1040 patients with type 2 diabetes in eastern Iran. Participants was selected by proportional stratified sampling method. The validity of DHLS was investigated through qualitative face validity, qualitative content validity, and structural validity (exploratory factor analysis and confirmatory factor analysis). The reliability of DHLS was checked by Cronbach's alpha coefficient, McDonald omega coefficient, and test–retest.

**Results:**

In exploratory factor analysis, 3 factors with eigenvalues greater than 1 were extracted, explaining 68.57% of the variance. These factors entered the confirmatory factor analysis, none of the questions were removed, and all questions had factor loading above 0.4. Cronbach's alpha coefficient and McDonald omega coefficient of DHLS were 0.919 and 0.922. Also, the Intraclass correlation coefficient of DHLS was 0.957. Finally, the DHLS was approved with 14 questions and the three subscales of Informational Health Literacy (6 items), Numerate Health Literacy (5 items), and Communicative Health Literacy (3 items).

**Conclusions:**

DHLS with 14 questions and the three subscales is a valid and reliable tool for examining diabetes health literacy in people with type 2 diabetes.

## Background

The increase in diabetes is a fundamental problem in healthcare systems around the world, and diabetes is considered one of the most challenging and highest chronic diseases [1, 2]. It's predicted that the total number of people with diabetes will reach 643 million by 2030 and by 2045 to 783 million [1]. The tenth leading cause of death in Iran is diabetes [3]. Currently, the prevalence of diabetes in the general Iranian population is 2–3%, while the prevalence in people over the age of 30 is 7% [3]. Side effects of diabetes often cause high financial costs and reduced quality of life, and the care and treatment of diabetes patients accounts for about 4% of the health budget and estimated that the medical cost of a patient diabetes is 2 to 5 times more than healthy people [4].

Although there is no definitive treatment, it is possible to prevent and manage the type 2 diabetes [5–7]. Health literacy is one of the most influential factors in controlling and preventing diabetes [5]. The World Health Organization has identified health literacy as one of the biggest determinants of health [8]. It has also advised the countries of the world to create a community to promote health literacy in different societies [8]. Health literacy refers to cognitive and social skills that include the motivation and ability of individuals to achieve the perception and use of information to maintain and improve their health [9].

Studies have shown that low health literacy can have adverse effects on chronic disease, so there is a need to improve individuals' health literacy levels to manage their health and make health decisions [10–12]. The results of some studies in Iran showed that most diabetic patients do not have sufficient health literacy [13, 14]. People with low health literacy in the management of diseases such as diabetes are less successful in implementing self-care behaviors. Diabetic patients must have the necessary knowledge and awareness of self-care behaviors, and health literacy plays an important role in the control of diabetes [15, 16].

To examine health literacy in patients with diabetes, a proper instrument is needed. In Iran, several measures (such as HELIA and TOHFLA) are used to assess health literacy [17, 18], but these instruments examine general health literacy and are not specifically designed for patients with type 2 diabetes. One of the important stages of any research is data collection, which requires the use of appropriate tools [19]. To examine the status of diabetes health literacy and to design effective intervention programs, it is necessary to design and assess diabetes-specific tools. Therefore, it is necessary to create specialized tools to investigate the health literacy of people with diabetes.

The aim of this study was to examine the psychometric properties of Persian version of diabetes health literacy scale (DHLS) among type 2 diabetic patients. This scale was designed for people with diabetes and approved by Lee [20]. The purposes of this study were to:

1. Translating and determining the cultural adaptation of the DHLS in patients with type 2 diabetes.
2. Determine of qualitative face validity, qualitative content, and structural validity (using exploratory factor analysis and confirmatory factor analysis) of the DHLS in patients with type 2 diabetes.
3. Determine the reliability of the DHLS in patients with type 2 diabetes.

## Methods

### Design and participants

The purpose of this cross-sectional study was to investigate the psychometric properties of the Persian version of DHLS in 1040 patients with type 2 diabetes in eastern Iran in 2021.

### Sample*** size***

To perform structural validity (exploratory factor analysis and confirmatory factor analysis), the sample size of 100 is weak, 200 is relatively good, 300 is good, 500 is very well, and 1000 and more is considered excellent [21, 22]. As recommended exploratory factor analysis (EFA) and confirmatory factor analysis (CFA) are not performed on the same dataset as this yields high danger of overfitting [23]. So, in this study, EFA was performed on 300 participants and CFA was performed on 1040 participants.

### Sampling method

The sampling method in this study was proportional stratified sampling method. Initially, the number of health centers and the population of each center in three cities in eastern Iran were determined (Cities were selected by random method). In the next step, in each city, each health center was considered as a stratum and the sample size was determined based on the population of each class. In the following, samples from each center were randomly selected from patients who met the inclusion criteria.

The inclusion criteria in this study were people with type 2 diabetes disease based on laboratory results, had type 2 diabetes for more than a year, and had a tendency to participate in this study and fill out an informed consent form. Questionnaires with incomplete information were removed in the data analysis step.

### Instruments

1. Demographic questionnaire: This questionnaire includes questions such as sex, age, job status, marital status education level, age of onset of diabetes, and duration of the diabetes.
2. Diabetes health literacy scale (DHLS): This scale consists of 14 questions, and three subscales of Informational Health Literacy (7 items), Numerate Health Literacy (4 items), and Communicative Health Literacy (3 items). Questions of this scale were measured with a five-option Likert scale (not really = 1, slightly = 2, moderately = 3, quite a lot = 4, very much = 5), and higher scores on the DHLS and each subscale indicate better health literacy status. This questionnaire was designed and confirmed by Lee, and the validity of the scale have been verified by EFA and CFA. In Lee study, the Cronbach's alpha coefficient of total scale and three subscales of Informational Health Literacy, Numerate Health Literacy, and Communicative Health Literacy were equal to 0.91, 90, 0.80, and 0.85, respectively. Also, the Intraclass correlation coefficient of total DHLS and three subscales of Informational Health Literacy, Numerate Health Literacy, and Communicative Health Literacy were equal to 0.89, 0.85, 0.85, and 0.80, respectively [20].

### Translation and cultural adaptation

First, the consent of the main designer of the questionnaire was obtained. In the first step, the English version of the questionnaire was translated into Persian by two experts. In the following, we reviewed two versions of the translated questionnaire and created the Persian version of the questionnaire. In the second step, the Persian version of questionnaire was translated into English by two experts. After reviewing the two versions, an English version of the questionnaire was produced. In the third stage, the English version of the questionnaire was compared with the original version of the questionnaire.

### Validity assessment

When the standard questionnaire is used and translated, quantitative face validity and quantitative content validity are not required to evaluate the psychometric standard questionnaire [24]. In this study, due to the use of a standard questionnaire, the validity of the questionnaire was investigated only by qualitative face validity and qualitative content validity.

### Face and content validity

To examine the qualitative face validity, the questionnaire was provided to a number of target groups and the questionnaire was investigated in terms of ambiguity, relevance, suitability and difficulty of each question and finally the required modification were taken. To examine the qualitative content validity, the questionnaire was given to 9 specialists in public health and health education and the questionnaire was investigated in terms of grammar, the use of appropriate words, the importance of items, time required to answer each question, placement of items in the proper place, and finally the required modification were taken.

### Structural validity

#### EFA

Before the conducting of EFA, Kaiser–Meyer–Olkin (KMO) test and Bartlett’s test of Sphericity were used to check adequacy of the sample and the suitability of data [25, 26]. In the EFA stage, the minimum factor loading of 0.4, eigenvalues more than 1, and scree plot were used to explore the number of potential latent factors [27, 28]. When the identified factors was explain at least of 60% of the variance, the results of EFA was consider acceptable [29, 30].

#### CFA

In CFA stage, at first, the Mahalanobis statistical index was used for assessed the outlier’s data and then, skewness and kurtosis were used for evaluating the data normality. The following indicators were used to assess goodness-of-fit of the model. These indicators consist of root mean square error of approximation (RMSEA),chi-square ratio to degree of freedom (× 2/df), parsimony comparative fit index (PCFI), parsimonious normed fit index (PNFI), goodness of fit index (GFI), adjusted goodness of fit index (AGFI), incremental fit index (IFI), comparative fit index (CFI), relative fit index (RFI), normed fit index (NFI), and parsimony goodness-of-fit index (PGFI) [31–33]. Standard goodness-of-fit indexes included χ2/df < 5, RMSEA < 0.08, AGFI > 0.8, PCFI > 0.5, PGFI > 0.5, PNFI > 0.5, and indices of GFI, CFI, IFI, RFI, GFI, and NFI greater than 0.9 [31–34].

#### Reliability assessment

In this study, Cronbach's alpha coefficient, McDonald omega coefficient, and test–retest were used to evaluate the reliability of the questionnaire. Results reported the Cronbach’s alpha coefficient of ranging from 0.70 to 0.95 is good [35, 36]. Also, the Intraclass Correlation Coefficient (ICC) was used to assess test–retest. The amount of ICC higher than 0.80 is acceptable [37]. To review the reliability, questionnaires were provided to 30 participants. Also, to review the test–retest, the questionnaire was given to the participants twice (the second phase was completed after 2 weeks).

#### Data analysis

In this study, EFA was performed using SPSS_V.20_ software. At this stage, the factors extracted in the EFA stage were examined by using AMOS _V.24_ software. Also, Pearson correlation was used to investigate the correlation between DHLS factors. The Cronbach’s alpha coefficient and McDonald’s omega coefficient were calculated using the SPSS_v20_ software and JASP_V. 0.11.1_ software, respectively.

## Results

### Descriptive characteristics

The mean (± standard deviation) age of participants in this study was 52.63 (± 14.70). The mean (± SD) age of onset of diabetes and duration of the diabetes were 43.58 (± 9.62) and 8.69 (± 6.80), respectively. The majority of participants in this study were men (n = 523, 50.3%) and married (n = 619, 59.6%). Most of the education level of participants were high school/diploma (n = 315, 30.3%) and middle school (n = 248, 23.8%). The job status of the majority of participants were housewives (n = 421, 40.6%) and self-employed (n = 238, 22.9%) (Table 1).

**Table 1:** Frequency distribution of demographic characteristics (n = 1040)

Variables		N	%
Sex	Men	523	50.3
	Women	517	49.7
Marital status	Married	619	59.6
	Single	420	40.4
Education level	Illiterate	30	2.9
	Elementary school	167	16.1
	Middle school	248	23.8
	High school/diploma	315	30.3
	Associate or bachelor's degree	229	22.0
	Master's degree or high degree	51	4.9
Job	Housewife	421	40.6
	Employed	123	11.8
	Self-employed	238	22.9
	Unemployed	39	3.8
	laborer	88	8.5
	Retired	129	12.4

### Validity assessment

First, the translation and cultural adaptation process of the questionnaire was carried out. Then, the questionnaire was checked using qualitative face validity and qualitative content validity, and four questions were modified.

#### EFA

**Fig. 1:**
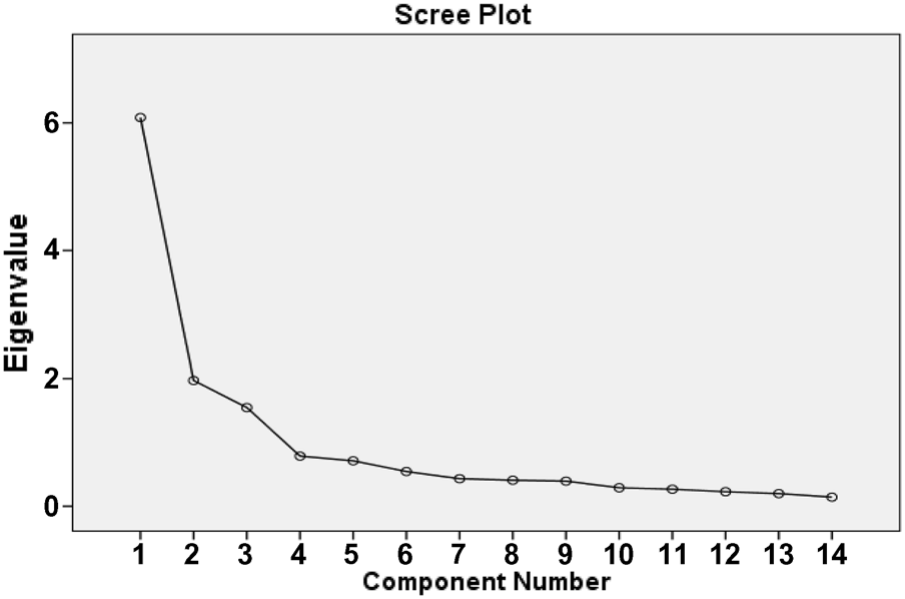
Scree plot of the factor analysis of the Persian version of DHLS

First, the data were analyzed using EFA. The results of KMO and Bartlett's Test of Sphericity showed that the sample size was sufficient for this section (KMO = 0.877, Bartlett’s test: p < 0.001, χ^2^ = 2508.555, df = 91). Also, based on the results of EFA, 3 factors with eigenvalues greater than 1 were extracted, explaining 68.57% of the variance. These factors were similar to the original questionnaire factors. Only in this study, a question (Question 7: When a change occurs in my personal plan, I can change the appointment date or time for a medical checkup) moved from factor 1 (F1: Informational health literacy) to factor 2 (F2: Numerate health literacy) (Tables 2, 3; Fig. 1).

**Table 2:** The three-factor structure of the Persian version of DHLS

Total variance explained									
Component	Initial Eigenvalues			Extraction sums of squared loadings			Rotation sums of squared loadings		
	Total	% of variance	Cumulative %	Total	% of variance	Cumulative %	Total	% of variance	Cumulative %
1	6.083	43.452	43.452	6.083	43.452	43.452	4.197	29.978	29.978
2	1.970	14.074	57.526	1.970	14.074	57.526	3.343	23.880	53.858
3	1.546	11.043	68.568	1.546	11.043	68.568	2.059	14.710	68.568
4	0.786	5.618	74.186						
5	0.712	5.084	79.270						
6	0.544	3.888	83.157						
7	0.432	3.085	86.242						
8	0.407	2.910	89.152						
9	0.394	2.814	91.966						
10	0.290	2.070	94.036						
11	0.267	1.911	95.947						
12	0.227	1.624	97.571						
13	0.198	1.413	98.983						
14	0.142	1.017	100.000						

Extraction method: principal component analysis

**Table 3:** Rotated factor matrix of the Persian version of DHLS

Rotated component matrix^a^			
Items	Component		
	F1: Informational health literacy	F2: Numerate health literacy	F3: Communicative health literacy
**T1**	**0.742**	0.264	0.004
**T2**	**0.884**	0.143	0.123
**T3**	**0.902**	0.134	0.148
**T4**	**0.842**	0.262	0.099
**T5**	**0.815**	0.049	0.255
**T6**	**0.603**	0.521	0.105
**T7**	0.348	**0.710**	0.183
**T8**	0.256	**0.616**	0.305
**T9**	0.162	**0.864**	0.051
**T10**	0.192	**0.776**	0.186
**T11**	0.018	**0.773**	0.008
**T12**	0.021	0.224	**0.741**
**T13**	0.212	0.076	**0.840**
**T14**	0.136	0.083	**0.721**

Extraction method: principal component analysis

^a^Rotation method: Varimax with Kaiser Normalization

a. Rotation converged in 5 iterations

#### CFA

**Fig. 2:**
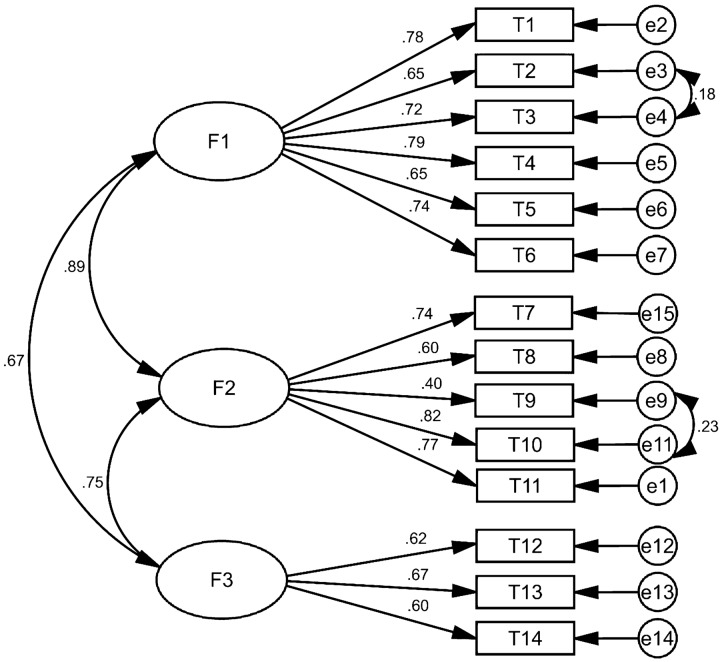
Standardized parameter estimates for the factor structure of the questionnaire of diabetes health literacy scale (F1: Informational health literacy; F2: Numerate health literacy; F3: Communicative health literacy)

In this section, the factors extracted in the EFA stage were evaluated by CFA. All goodness-of-fit indexes had a standard rate (for example: χ^2^/df = 4.604, RMSEA = 0.059, GFI = 0.955, CFI = 0.959) and the final model was confirmed with three factors and 14 questions (Table 4). At this stage, none of the questions were removed and the factor lodging of all questions were above 0.4 (Table 5, Fig. 2).

**Table 4:** The model fit indicators of the Persian version of DHLS

Goodness of fit indices	Confirmatory factor analysis	Acceptable value
χ^2^	331.470	–
df	72	–
X^2^/df	4.604	< 5
p-value	0.000	p > 0.05
CFI	0.959	> 0.9
GFI	0.955	> 0.9
RMSEA	0.059	< 0.08
IFI	0.959	> 0.9
RFI	0.934	> 0.9
NFI	0.948	> 0.9
PNFI	0.750	> 0.5
PCFI	0.758	> 0.5
PGFI	0.655	> 0.5
AGFI	0.934	> 0.8

**Table 5:** Factor loadings of the Persian version of DHLS

Subscales	Items	Factor loadings
F1: Informational health literacy	T1: I can read and understand booklets and educational materials related to diabetes	0.778
	T2: I can understand the written information given by the physician about diabetes treatment or an examination	0.646
	T3: I can receive and print the results of my diabetes test through the website that has been announced by the lab, hospital, etc	0.721
	T4: I can understand the information about diabetes that I sought from different sources (for example booklets, TV, Internet, etc.)	0.786
	T5: I understand the information provided by the health-care provider on diabetes management	0.651
	T6: I can earn reliable information about diabetes from different sources	0.736
F2: Numerate health literacy	T7: When a change occurs in my personal plan, I can change the appointment date or time for a medical checkup	0.741
	T8: I can calculate the next time taking my diabetes medications	0.602
	T9: I can determine the amount of carbohydrate content per meal from the nutrition label on food packaging	0.400
	T10: Based on the results of my blood glucose test, I can understand whether my blood glucose levels are normal or not	0.822
	T11: I can understand information about diabetes that are provided as ratios, probabilities or graphs	0.772
F3: Communicative health literacy	T12: When I have a question about diabetes, I usually ask a health-care provider	0.616
	T13: I can explain the condition of my diabetic disease for health care provider	0.673
	T14: When I eating out with my friends or colleagues, I can explain the reason why I should have a diabetic diet	0.596

#### Reliability assessment

Cronbach's alpha coefficient and McDonald omega coefficient of DHLS were 0.919 and 0.922. Cronbach's alpha coefficient of Informational health literacy (F1), Numerate health literacy (F2), and Communicative health literacy (F3) were 0.865, 0.879, and 0.784, respectively. McDonald omega coefficient of Informational health literacy (F1), Numerate health literacy (F2), and Communicative health literacy (F3) were 0.871, 0.881, and 0.800, respectively. The ICC of DHLS was 0.957 (Table 6). The results of Pearson correlation analysis showed that there was a significant positive correlation between the factors of DHLS (Table 7).

**Table 6:** Descriptive statistics of the Persian version of DHLS

Subscales	Item	Range	Cronbach’s alpha coefficients	McDonald’s omega coefficients	Intraclass correlation coefficient (ICC)	95% confidence interval		P-value
						Lower bound	Upper bound	
Factor 1: Informational health literacy	6	6–30	0.865	0.871	0.976	0.950	0.989	< 0.001
Factor 2: Numerate health literacy	5	5–25	0.879	0.881	0.921	0.834	0.962	< 0.001
Factor 3: Communicative health literacy	3	3–15	0.784	0.800	0.911	0.813	0.957	< 0.001
Total diabetes health literacy scale (DHLS*)*	14	14–70	0.919	0.922	0.957	0.910	0.980	< 0.001

**Table 7:** Pearson correlation between DHLS subscales

Subscales	Informational health literacy	Numerate health literacy	Communicative health literacy
Informational health literacy	1		
Numerate health literacy	0.678*	1	
Communicative health literacy	0.513*	0.500*	1

^*^Correlation is significant at the 0.01 level (2-tailed)

## Discussion

A key issue to consider when considering health literacy tools is the range of concepts that need to be measured. According to a recent systematic review of measurement characteristics [38], previously reported instruments for measuring the health literacy of diabetic patients measure limited ranges of basic skills (such as reading and comprehension), with the exception of the Health Literacy Scale [39] and the Health Literacy Questionnaire [40]. Although these two scales measure more than basic skills, they have been criticized for their inability to measure counting. The Health Literacy Questionnaire contains 44 items, making it less likely to use it in Clinical environments. Unlike existing tools, the DHLS assessed in this study includes three dimensions of health literacy, including Informational Health Literacy, Numerate Health Literacy, and Communicative Health Literacy. In other words, health professionals can use DHLS to assess their patients' diabetes health literacy more comprehensively. Evaluated levels of three dimensions of health literacy can be used to adapt information education for diabetics and thus optimize educational outcomes.

The main purpose of this study was to evaluate the psychometric properties of the new Persian version of DHLS using a sample of patients with type 2 diabetes in Iran. This study translated and validated measures the Diabetes Health Literacy Questionnaire for the first time in Iran. Conceptually comparable to the original DHLS, which was evaluated on Korean patients with type 2 diabetes [20], we found that it had excellent psychometric properties with high reliability and excellent convergence properties as well as factorial validity.

In our study, the overall internal consistency (Cronbach's alpha coefficient) of the Persian version of DHLS was excellent. Due to the consistency of internal consistency, Cronbach's alpha increases when the scale contains more items [41]. Although DHLS is a relatively short tool, in this study, Cronbach's alpha for DHLS was exceeded 0.8. This means that the items in each DHLS subscale measure exactly the same underlying attribute [42].

## Strength and limitations

One of the limitations of this study was the COVID-19 pandemic, which led to a slow process of data collection. Another limitation of this study was that the information was completed in self-reports, which may be had some errors. The first strength of this study was that the psychometric process was performed by the face validity, content validity, structure validity (EFA and CFA), and reliability (Cronbach’s alpha coefficient, McDonald’s omega coefficient and ICC). The second strength of this study was the large sample size. The third strength of this study was that the samples were selected from three different cities.

## Conclusion

Finally, in this study, the DHLS was approved with 14 questions and the three subscales of Informational Health Literacy (6 items), Numerate Health Literacy (5 items), and Communicative Health Literacy (3 items). The Persian version of DHLS is a valid and reliable tool for measuring the health literacy status in in patients with type 2 diabetes in Iran.

## Data Availability

All data generated or analysed during this study are included in this published article.

## Abbreviations

DHLS: Diabetes health literacy scale
EFA: Exploratory factor analysis
CFA: Confirmatory factor analysis
KMO: Kaiser–Meyer–Olkin
F1: Informational health literacy
F2: Numerate health literacy
F3: Communicative health literacy
RMSEA: Square error of approximation
x2/df: Chi-square ratio to degree of freedom
PCFI: parsimony comparative fit index
PNFI: Parsimonious normed fit index
GFI: Goodness of fit index
AGFI: Adjusted goodness of fit index
IFI: Incremental fit index
CFI: Comparative fit index
RFI: Relative fit index
NFI: Normed fit index
PGFI: Parsimony goodness-of-fit index
