# Correction: Psychometric properties of Persian version of diabetes health literacy scale (DHLS) in patients with type 2 diabetes

**DOI:** 10.1186/s13098-022-00923-9

**Published:** 2022-11-17

**Authors:** Mahdi Moshki, Ali Alami, Zohreh Zadehahmad, Mousa Ghelichi-Ghojogh, Mitra Dogonchi, Alireza Jafari

**Affiliations:** 1 Department of Health Education and Health Promotion, School of Health, Social Determinants of Health Research Center, Gonabad University of Medical Sciences, Gonabad, Iran; 2 Department of Epidemiology and Bio-Statistics, School of Public Health, Social Determinants of Health Research Center, Gonabad University of Medical Sciences, Gonabad, Iran; 3 Department of Public Health, School of Health, Torbat Heydariyeh University of Medical Sciences, Torbat Heydariyeh, Iran; 4 Health Management and Social Development Research Center, Faculty of Health, Golestan University of Medical Sciences, Gorgan, Iran

## Correction: Diabetology & Metabolic Syndrome (2022) 14:139 10.1186/s13098-022-00910-0

Following publication of the original article [1], the author identified an error in the first author’s affiliation.

- The incorrect affiliation:
- Mahdi Moshki
- Department of Health Education and Health Promotion, School of Health, Social Development and Health Promotion Research Center, Gonabad University of Medical Sciences, Gonabad, Iran
- The correct affiliation for first author:
- Mahdi Moshki
- Department of Health Education and Health Promotion, School of Health, Social Determinants of Health Research Center, Gonabad University of Medical Sciences, Gonabad, Iran

The original article [1] has been revised.
